# Comparative Histological and Transcriptional Analysis of Maize Kernels Infected with *Aspergillus flavus* and *Fusarium verticillioides*

**DOI:** 10.3389/fpls.2017.02075

**Published:** 2017-12-06

**Authors:** Xiaomei Shu, David P. Livingston, Charles P. Woloshuk, Gary A. Payne

**Affiliations:** ^1^Department of Entomology and Plant Pathology, North Carolina State University, Raleigh, NC, United States; ^2^Department of Crop Science, North Carolina State University, Raleigh, NC, United States; ^3^Department of Botany and Plant Pathology, Purdue University, West Lafayette, IN, United States

**Keywords:** maize kernel, *Aspergillus flavus*, *Fusarium verticillioides*, histology, RNA-sequencing

## Abstract

*Aspergillus flavus* and *Fusarium verticillioides* infect maize kernels and contaminate them with the mycotoxins aflatoxin, and fumonisin, respectively. Genetic resistance in maize to these fungi and to mycotoxin contamination has been difficult to achieve due to lack of identified resistance genes. The objective of this study was to identify new candidate resistance genes by characterizing their temporal expression in response to infection and comparing expression of these genes with genes known to be associated with plant defense. Fungal colonization and transcriptional changes in kernels inoculated with each fungus were monitored at 4, 12, 24, 48, and 72 h post inoculation (hpi). Maize kernels responded by differential gene expression to each fungus within 4 hpi, before the fungi could be observed visually, but more genes were differentially expressed between 48 and 72 hpi, when fungal colonization was more extensive. Two-way hierarchal clustering analysis grouped the temporal expression profiles of the 5,863 differentially expressed maize genes over all time points into 12 clusters. Many clusters were enriched for genes previously associated with defense responses to either *A. flavus* or *F. verticillioides*. Also within these expression clusters were genes that lacked either annotation or assignment to functional categories. This study provided a comprehensive analysis of gene expression of each *A. flavus* and *F. verticillioides* during infection of maize kernels, it identified genes expressed early and late in the infection process, and it provided a grouping of genes of unknown function with similarly expressed defense related genes that could inform selection of new genes as targets in breeding strategies.

## Introduction

*Aspergillus flavus* is an opportunistic fungal pathogen that can grow either as a saprophyte in the soil or as a pathogen of many plant species. Hosts of *A. flavus* include maize kernels, peanuts, cottonseeds and tree nuts (Payne, 1992; Payne and Yu, 2010; Scheidegger and Payne, 2003; St. Leger et al., 2000). Unlike *A. flavus, Fusarium verticillioides* can colonize maize seeds as an endophyte or necrotroph. Under certain conditions, often related to plant stress, *F. verticillioides* can cause seedling blight, ear rot, and stalk rot (Bacon et al., 1992; Munkvold, 2003; Pei-Bao et al., 2010).

*Aspergillus flavus* and *F. verticillioides* are capable of invading kernels in the field by several routes. *F. verticillioides* can grow from infected seeds, colonize plant stalks, and grow into ears and infect kernels (Bacon et al., 1992; Duncan and Howard, 2010). While this route of kernel infection is important, kernels are infected more commonly in the field from airborne inoculum, which infects silks and grows down the silk channel into the ear (Munkvold, 2003). *A. flavus* also colonizes maize silks and grows down the silk channel into the ear (Marsh and Payne, 1984; Smart et al., 1990). Both fungi can colonize and produce mycotoxins in kernels without visible damage (Munkvold et al., 1997; Widstrom et al., 1981) but insect damage and other wounds provide sites for infection and lead to higher levels of mycotoxin contamination (Koehler, 1942; Lillehoj et al., 1975; Sobek and Munkvold, 1999). Effective management of these two diseases must account for the role of insects in the disease.

Effective resistance to either of these two fungi in commercial maize lines has been difficult to achieve, due in part to the quantitative nature of resistance, large environment effects on the diseases, and the lack of characterized genes for host resistance (Payne et al., 1986; Munkvold, 2003; Dolezal et al., 2014; Shu, 2014; Warburton and Williams, 2014; Lanubile et al., 2015). Further complicating the identification of resistance genes is a poor understanding of the temporal pattern of gene expression during the colonization of multiple tissue types within seeds, each of which may differ in their expression profiles. Better characterization of resistance gene expression at stages in the infection process could indicate optimum times for resistance evaluation.

This study was directed at the identification of defense-related genes expressed in maize kernels at several stages during colonization by *A. flavus* and *F. verticillioides*. By observing fungal colonization and measuring gene expression in maize seeds from 4 to 72 h post inoculation (hpi) we were able to identify genes expressed early in pathogenesis as well as those expressed during the colonization of specific tissue types of the kernel. The study also allowed analysis of similarities and differences in response of maize seeds to these two pathogens with different tropic behaviors. We are unaware of any study that compares both histological examination and gene profiling of these two fungi under the same growing conditions or one that has examined very early gene expression in maize in response to these fungi.

Large-scale RT-qPCR, RNA-seq, microarray and proteomic studies have been conducted to dissect maize transcriptional changes during infection by *A. flavus* (Chen et al., 2002; Dolezal, 2010; Luo et al., 2011; Pechanova et al., 2011; Kelley et al., 2012; Brown et al., 2013; Dolezal et al., 2013; Asters et al., 2014; Musungu et al., 2016). Results from these studies have shown that maize seeds respond to infection by the expression of characterized defense related genes. As an example, a recent study by Musungu et al. (2016) reported that maize JA, ET, and ROS pathways are associated with defense response to *A. flavus* infection. Similar classes of maize genes show elevated gene expression during infection by *F. verticillioides* (Lanubile et al., 2010, 2012, 2013, 2014; Kim et al., 2015; Wang et al., 2016). Wang et al. (2016) reported that maize ABA, JA and SA signaling pathways are associated with triggering immunity against *F. verticillioides.*

*Fusarium verticillioides* also has been shown to induce more drastic gene expression changes in the susceptible maize lines than in the resistant lines (Lanubile et al., 2010, 2013, 2014; Campos-Bermudez et al., 2013). The pathogenesis-related genes are also transcribed at higher levels in kernels of the *F. verticillioides*-resistant lines before infection (Lanubile et al., 2010, 2014; Campos-Bermudez et al., 2013). By using RNA-seq technology, Lanubile et al. (2014) found that the resistant maize line showed higher activation of genes involved in JA and ET signaling pathways and shikimate biosynthesis pathway. Lanubile et al. (2015) reported that higher gene expression and enzymatic activities were detected in maize kernels that are resistant to *A. flavus* as well as other two *Fusarium* spp., *F. proliferatum* and *F. subglutinans*. They also found that expression levels of *PR* genes remained higher in the resistant lines after inoculation (Lanubile et al., 2015).

The consistent observation that infection by *A. flavus* and *F. verticillioides* causes a change in expression of these upstream signaling molecules suggest that they may be good markers for a host resistance response to these fungi in developing maize seeds. The overall objectives of this study were to provide fundamental information on the colonization of maize seeds by these two fungi, determine the temporal profile of maize gene expression during infection, and identify genes with similar expression profiles to resistance associated genes.

## Materials and Methods

### Plant Material, Fungal Inoculation, and Sampling

**FIGURE 1:**
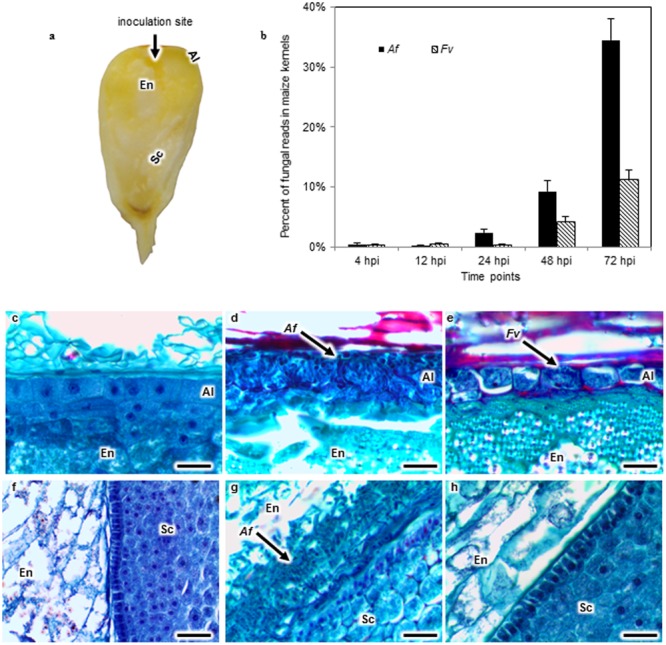
*Aspergillus flavus (Af)* and *Fusarium verticillioides (Fv)* colonization of maize kernels developing in the field. Kernels in early dough stage (20–22 days after pollination) were inoculated by inserting a pin dipped into either an *Af* or *Fv* conidial suspension into the crown of exposed kernels, introducing approximately 13 spores into the endosperm (En) tissue **(a)**. The percent of fungal RNA-seq reads in the total reads of *Af* or *Fv* infected kernels at 4, 12, 24, 48, and 72 h post inoculation (hpi) indicated that fungal tissues increased over time in the kernel, and *A. flavus* more extensively colonized the kernel than *F. verticillioides* at 24, 48, and 72 hpi **(b)**. Kernel sections collected at 72 hpi were stained with safranin and fast green **(c–h)**. No fungal colonization was observed in the aleurone (Al), En or scutellum (Sc) of a mock inoculated kernel **(c,f)**. Af colonized and destroyed the Al **(d)**, whereas *Fv* colonized around the intact Al **(e)**. *Af* colonized the EnSc interface forming a biofilm-like structure **(g)**. But the biofilm like structure was not observed in *Fv* inoculated kernels **(h)**. Arrows denote fungal colonization. Scale bars: 30 μm.

Fungal strains (*A. flavus* NRRL *3357, F. verticillioides n16*) were grown on potato dextrose agar (PDA) plates at 28°C for 5 days. Conidial suspensions were harvested by adding sterile distilled water containing 0.05% (v/v) Triton X-100 (Fisher) and scraping the plates using a glass spreader. The concentration of conidia was quantified using a hemocytometer and diluted to 1 × 10^6^ conidia/ml for inoculation. Maize inbred line B73 was grown at the Central Crops Research Station near Clayton, NC plots as described by Shu et al. (2015). Maize ears were hand-pollinated and covered with paper bags until inoculation at 21–22 days after pollination. Inoculation was conducted by wounding the kernel with a 3 mm needle bearing approximately 13 conidia (Dolezal et al., 2014; **Figure 1a**). Kernels for the mock treatment were inoculated with sterile distilled water containing 0.05% (v/v) Triton X-100.

For each treatment three ears randomly selected from the 70 plots were harvested at 4, 12, 24, 48, and 72 hpi. Each of the three ears was treated as a biological replication. At harvest kernels from ears collected at each time point were divided into two subsets. One subset of kernels at each of the time points was examined for tissue specific colonization by the fungi as described by Shu et al. (2015). The other subset of kernels at each time point was frozen in liquid nitrogen immediately and stored at -80°C until RNA extraction and sequencing by Illumina HiSeq.

### Tissue Fixation, Embedding, and Microscopy

Six kernels from each ear replicate were fixed and dehydrated using the protocol modified from Livingston et al. (2009, 2013) and Shu et al. (2015). Briefly, we used a modified FAA fixative consisting of 45% methanol, 10% formaldehyde and 5% glacial acetic acid. The paraffin blocks were sectioned with a RM2255 microtome (Leica) and mounted on slides (Gold Seal). Slides were dried on a hot plate overnight and stored at room temperature. Paraffin was removed by dipping the slides in 100% xylene. Sections were then rehydrated with an ethanol series. Safranin and fast green staining were applied to differentiate tissue structure of maize kernels and the fungus grown in the kernel (**Figures 1c–h**). The rehydrated sections were stained with safranin, dehydrated with an ethanol series, and counter stained with fast green (Fisher) (Shu et al., 2015). Stained sections were mounted in permount mounting medium (Fisher) and covered with coverslips. Images of stained tissues were collected on an Eclipse E600 light microscope (Nikon). Images were captured on an Infinity1-3C digital camera, and analyzed with the software Infinity Analyze (Lumenera).

### RNA-Isolation, Illumina Library Preparation, and Sequencing

Eight frozen kernels from each ear replicate were pooled and ground in liquid nitrogen with a mortar and pestle. About 100 mg of ground tissue was added to 0.75 ml of saturated phenol, pH 6.6 (Fisher), and homogenized for 2 min. Samples were then dissolved in Tris EDTA buffer, pH 8.0 (ACROS Organics), extracted with 5:1 acid phenol: chloroform, pH 4.5 (Fisher), and precipitated with ice-cold 100% ethanol overnight. Total RNA was further purified with an RNeasy Mini Kit (Qiagen) according to the manufacturer’s instructions. The quality and concentration of RNA was analyzed using an RNA Pico chip on an Agilent Bioanalyzer. The cDNA library construction and sequencing runs on an Illumina HiSeq were done by the Genomic Sciences Laboratory, North Carolina State University. Multiple samples with different barcodes were loaded in three lanes and sequenced to obtain 100 bp single-end reads.

### RNA-Seq Data Analysis

Illumina reads were sorted by barcodes, and adapter sequences were trimmed. The raw sequencing reads were then analyzed using the iPlant Collaborative Discovery Environment (Matasci and McKay, 2013). Reads of the same individual from multiple lanes were concatenated using the software named ‘Concatenate Multiple Files.’ The quality of the reads was checked using FastQC 0.10.1 (data not shown) and then aligned to the maize genome (Ensembl 14) by using TopHat2-SE (Trapnell et al., 2012). Maize transcripts were assembled using Cufflinks2. All transcripts from Cufflinks output were merged into a single transcriptome annotation file using Cuffmerge2 and used for Cuffdiff2. The TopHat2-SE output files were sorted using SAMtools sort BAM file before conducting Cuffdiff2 analysis. Gene expression levels were analyzed using Cuffdiff2 (Trapnell et al., 2012).

We assigned the differentially expressed maize genes (*q*-value < 0.05 and fold change > 2) into functional categories using MapMan (Usadel et al., 2009). Then we used the MaizeSequence database to obtain gene annotation (Schnable et al., 2009). For the genes without annotation in MaizeSequence database, we combined a few databases, including MaizeGDB, ProFITS, GRASSIUS, CoGePedia, Maize Protein Atlas databases (Lawrence et al., 2004; Lyons and Freeling, 2008; Yilmaz et al., 2009; Ling et al., 2010; Castellana et al., 2013) and the *R-like* gene database published by Song et al. (2015). Differentially expressed maize genes were also placed into biosynthetic and regulatory pathways using the MaizeCyc database and the bioinformatics software Biocyc (Caspi et al., 2012). Clustering was performed using JMP, 11 (SAS Institute Inc., Cary, NC, United States).

### Data Deposition

RNA-seq data generated in this study were deposited in the National Center for Biotechnology Information (NCBI) sequence read archive (SRA) collection, accession number PRJNA418364. The *de novo* assemblies of transcriptomes and assembled sequences together with sequence annotation were posted on figshare^1^.

## Results

**FIGURE 2:**
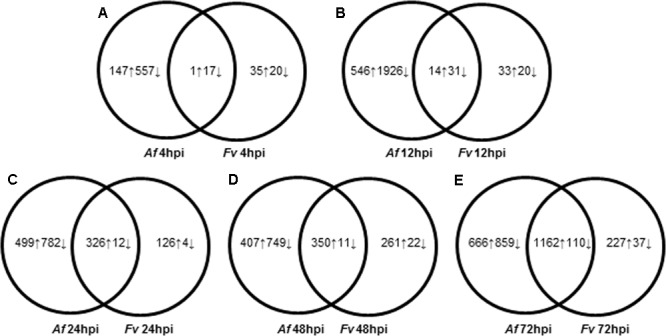
Dynamic changes of kernel transcriptome during *A. flavus* (*Af*) and *F. verticillioides* (*Fv*) infection. **(A–E)** Show total numbers of maize genes differentially expressed in response to *A. flavus* and *F. verticillioides* infection at 4 **(A)**, 12 **(B)**, 24 **(C)**, 48 **(D)**, and 72 **(E)** hours post inoculation (hpi). Total numbers of up (↑) and down (↓)-regulated genes of treatment-specific and shared between treatments are displayed in Venn diagrams.

**FIGURE 3:**
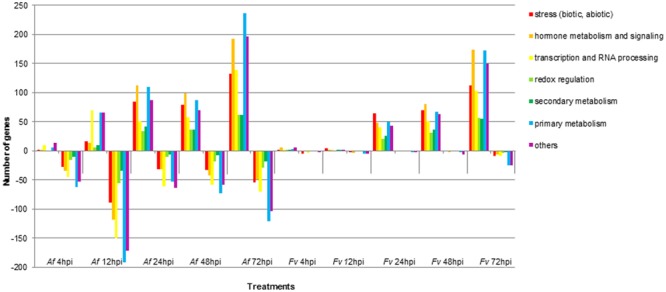
Functional categories of maize genes differentially regulated upon infection by *A. flavus* (*Af*) and *F. verticillioides* (*Fv*) at different time points. Colored bars represent the numbers of regulated genes with annotated function. Positive bars denote numbers of up-regulated genes. Negative bars denote numbers of down-regulated genes.

Maize kernels developing in the field were inoculated with either *A. flavus* or *F. verticillioides* (**Figure 1a**) and colonization followed by histological examination of stained kernel sections at 4, 12, 24, 48, and 72 hpi (**Figures 1c–h**). RNA was isolated from a subsample of kernels taken at each of these time points and subjected to RNA-seq analysis. This paired analysis on the same tissue samples permitted a direct association of tissue colonization with global gene expression in maize kernels in response to infection by these two fungi (**Figures 2, 3**).

### Colonization of Maize Kernels by *A. flavus* and *F. verticillioides*

Histological sections from kernels collected at 4, 12, or 24 hpi did not reveal fungal mycelia. In contrast, extensive colonization by *A. flavus* and *F. verticillioides* was observed at 48 hpi in the aleurone and the outermost layer of the endosperm (**Figures 1d,e**). Disruption of the aleurone was associated with the presence of *A. flavus* mycelium (**Figure 1d**), whereas colonization of the aleurone tissue by *F. verticillioides* at 48 hpi was less extensive and the cells appeared intact (**Figure 1e**). By 72 hpi, both pathogens had colonized the aleurone and endosperm. Mycelia of *A. flavus*, but not that of *F. verticillioides*, was found in the embryo at 72 hpi. *A. flavus* formed a dense mat of mycelium at the interface of the endosperm and scutellum of the embryo (**Figure 1g**). The scutellum was rarely colonized prior to the formation of the mat. No such structure was observed in the *F. verticillioides* colonized kernels. Visual observation as well as transcript abundance from RNA-seq analysis indicated more extensive colonization of kernels by *A. flavus* than *F. verticillioides* at each sample time (**Figure 1b**).

### Temporal Expression of Maize Genes in Response to Fungal Colonization

Pathogenesis by *A. flavus* or *F. verticillioides* resulted in broad transcriptional and presumably large metabolic changes in maize kernels during infection. Expression analysis identified 5,863 maize genes differentially expressed at one or more of the time points during pathogenesis by these two fungi. The greatest number of maize genes differentially expressed in response to the two fungi occurred at 72 hpi. At each time point, more genes were differently expressed in response to *A. flavus* than to *F. verticillioides*. While *A. flavus* did colonize seeds more extensively than *F. verticillioides* (**Figure 1**), it is unlikely that differences in colonization alone resulted in greater transcriptional changes in maize kernels. Fungal biomass accumulation by *A. flavus* in maize seeds 48 hpi was similar to that of *F. verticillioides* biomass accumulation *at* 72 hpi. At these time points, 757 genes were upregulated and 760 genes were downregulated in response to *A. flavus* infection and 1389 genes were upregulated and 147 genes were downregulated in response to *F. verticillioides* infection. Furthermore, colonization of maize seed by *F. verticillioides* 72 hpi resulted in greater expression of more chitinase genes, stress-related genes, hormone metabolism and signaling genes, transcription and RNA processing, secondary metabolism and primary metabolism genes than colonization by *A. flavus* 48 hpi (**Figure 3** and Supplementary Table S1).

Monitoring maize gene expression at five time points allowed association of gene expression profiles with stages of fungal colonization. Unique and overlapping sets of maize genes were differentially expressed in maize kernels in response to the two pathogens over the entire infection process (**Figure 2** and Supplementary Table S1). Maize kernels recognized infection by *A. flavus* within 4 hpi and increased expression of 148 genes and decreased expression of 574 genes. Fewer maize genes were differentially expressed in response to *F. verticillioides* infection, with 36 maize genes up regulated and 37 down regulated at 4 hpi (**Figure 2**). At this sample point, both pathogens repressed the expression of *chitinase2* (*chn2*), *heat shock protein26*, a 22.0 kDa class IV heat shock protein, and a cell wall modification related gene. *Chitinase 2* (*chn2*) was elevated in response to either fungus 12, 24, 48, and 72 hpi. By 24 hpi, 231 genes with known function were more highly expressed in response to either fungus including a set of *PR* genes. Within 24 hpi, these fungi induced expression of maize genes assigned to all seven functional categories (**Figure 3** and Supplementary Table S1), including genes associated with stress, hormone metabolism and signaling, transcription and RNA processing, redox regulation, as well as primary and secondary metabolism. Continued colonization of maize seeds resulted elevated expression of more genes with known function. At 72 hpi, 724 genes with known function are more highly expressed in response to either fungus, including defense-related genes. Notably, *PR-4, PR-5, PR-10* and *PR-10.1* were consistently more highly expressed in response to either fungus at 24, 48, and 72 hpi. At 72 hpi, 63 genes with known function are more highly expressed in response to either fungus, including a PR protein (GRMZM2G178199).

Ward’s Two-way Hierarchal Clustering Analysis allowed us to cluster expression levels of the 5,863 differentially expressed genes by time and by genes. When clustered by time, early time points (4–12 hpi) clustered together and later time points (24–72 hpi) clustered together. RNA samples collected at 4, 12, and 24 hpi provided transcriptional data at stages of colonization before the fungi were observed by histological examination, whereas 48 and 72 hpi RNA samples provided data from more advanced stages of fungal colonization (**Figures 1b–h**).

Among the genes expressed early during infection were genes that have been used as markers for activation of host defenses in maize and other plants (Supplementary Table S1). In maize kernels infected with *A. flavus*, more *PR-like* genes were down regulated than up regulated at 4 and 12 hpi. There was no significant increase in expression of any *PR-like* genes in maize seed at 4 hpi in response to infection by either fungus. In contrast, expression of genes for *PR-1, PR-10*, three *chitinases*, and a *glucan endo-1,3-beta-glucosidase 7* significantly decreased after infection with *A. flavus*, and expression of *chitinase 2* (*chn2*) decreased in *F. verticillioides* infected kernels (Supplementary Table S1). By 12 hpi, expression of *PR-5* and *chitinase* 2 (*chn2*) were elevated in response to either fungus. By 24 hpi several *PR-like* genes were more highly expressed after infection by the two fungi, including *PR-10*. Overall, infection of maize by *A. flavus* resulted in a larger number of *PR-like* and *R-like* genes with decreased expression compared to *F. verticillioides* at 4 and 12 hpi.

In plants, both coiled-coil nucleotide-binding site leucine-rich repeat (CC-NBS-LRR) and leucine-rich repeat receptor-like kinase (LRR-RLK) receptors are R-like proteins important in the resistance response to pathogens. Of the 378 *R-like* genes predicted in the maize genome (Ling et al., 2010; Song et al., 2015), 40 of 151 predicted CC-NBS-LRR genes were differentially expressed during infection by *A. flavus* and/or *F. verticillioides*. Of the 227 predicted *LRR-RLK* genes in maize, 36 were differentially expressed during one or more time points. Expression of three of these genes decreased at 4 hpi upon infection by *A. flavus*. None of these 36 differentially expressed *LRR-RLK* genes showed decreased expression in kernels infected with *F. verticillioides* until 72 hpi. Some of the *LRR-RLKs* were differentially expressed at 24, 48, and 72 hpi during infection of either *A. flavus* or *F. verticillioides*.

**FIGURE 4:**
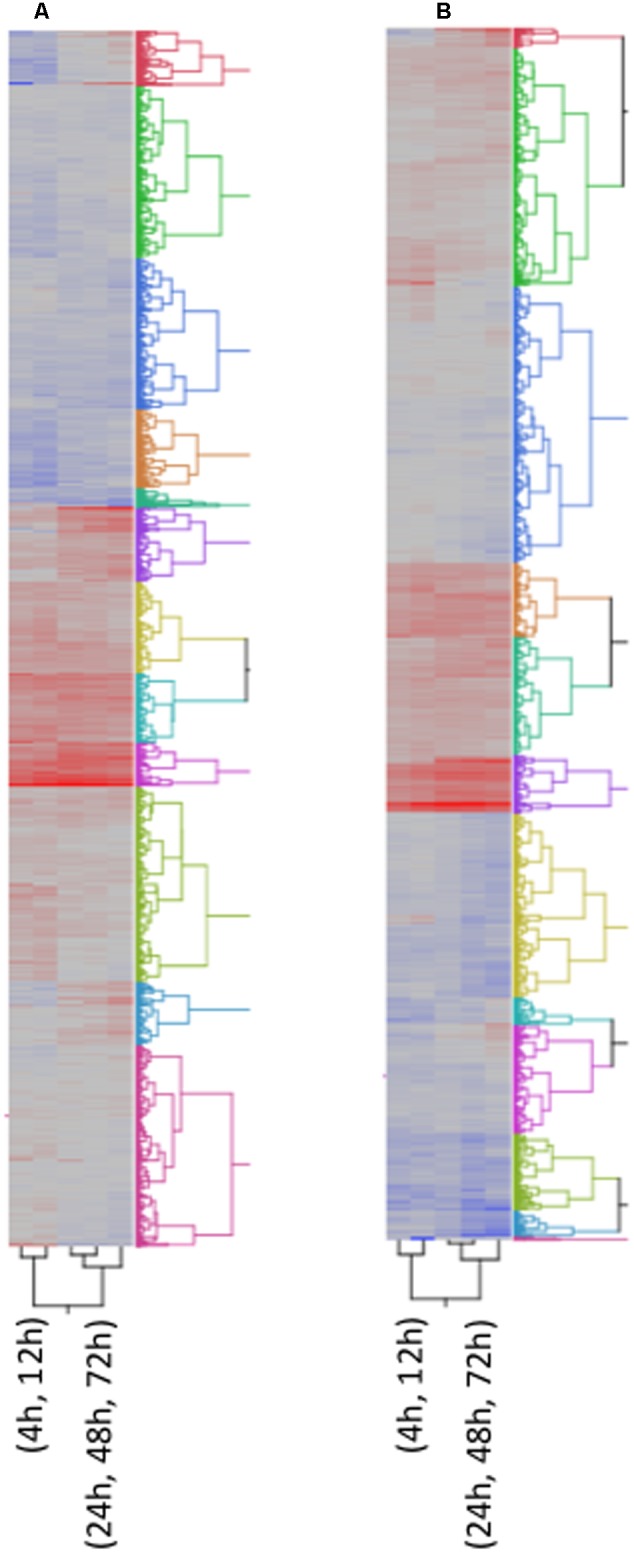
Two way Clustering of differentially expressed genes. FPKM values of differentially expressed genes were Ln transformed and Ward’s Hierarchical Clustering was performed. The number of clusters was set at 12 and clusters were color coded. The color map indicates relative gene expression over time with red indicating high expression and blue indicating low expression. **(A)** *A. flavus*; **(B)** *F. verticillioides*.

Plant defense to pathogen colonization is mediated by hormone signaling pathways. SA, JA, and ET govern the best described signaling pathways through their regulation of resistance gene expression, including *PR*-like genes. Because these two fungi are capable of different tropic interactions with the host, we predicted that maize seed would differ between the two fungi in the type or timing of gene expression. Expression of genes for SA biosynthesis increased from 24 to 72 hpi in response to *A. flavus* infection, but not until 72 hpi in response to *F. verticillioides*. Cluster analysis of gene expression data collected at the five time points in response to *A. flavus* infection placed the SA biosynthetic gene encoding the 3-phosphoshikimate 1-carboxyvinyltransferase gene into a cluster with a set of *PR-like* genes, including *PR-5* (**Figure 4** and Supplementary Table S1).

Several genes encoding maize 12-oxo-phytodienoic acid reductases (OPRs) and lipoxygenases (LOXs) were differentially expressed in maize seeds in response to either fungus (Supplementary Table S1). Expression of both *LOX4* and *LOX13* increased in response to *A. flavus* at 24 hpi and in response to *F. verticillioides* at 72 hpi. Two other *LOX* genes, *LOX10* and *LOX11*, were down regulated at 12 hpi in response to *A. flavus* infection, and *LOX10* was up regulated only at 24 hpi (Supplementary Table S1). These two genes were not differentially expressed in response to infection by *F. verticillioides*. Infection by *A. flavus* increased expression of *OPR2* at 24 and 48 hpi, and expression of *ORP2, ORP3*, and *ORP5* was up regulated in response to *A. flavus* at 48 hpi and to *F. verticillioides* at 72 hpi.

### Hierarchical Clustering of Genes Expression Profiles

Many of the maize genes differentially expressed in response to infection by *A. flavus* or *F. verticillioides* lack annotation or known biological function (Supplementary Table S1). To gain additional insight into possible involvement of these genes in resistance, we performed Ward’s Two-way Hierarchical Clustering (**Figure 4**) to group genes based on their profile of expression over time. This analysis allowed the clustering of genes with known or putative function with genes of unknown function. Clustering of unknown genes with resistance genes does not indicate a direct role for them in recognition and defense, but a similar profile of expression may indicate a role for these genes during different stages of disease development. This information may also guide future studies on sampling times to further characterize these genes.

**Figure 4** shows the clustering of all 5,863 genes into 12 clusters. The respective cluster membership for each gene is listed in Supplementary Table S1. The temporal expression of maize genes in response to these two fungi differed. This difference could be due to rate of growth or to differences in host response to the two species. For this reason, an independent cluster analysis of gene transcription was performed for each fungus (**Figures 4A,B**). Some clusters were enriched in defense related genes (**Figure 4** and Supplementary Table S1), for example clusters 6A and 10A associated with *A. flavus* infection (‘A’ denotes *A. flavus-*associated cluster). *A. flavus* Cluster 6A (Supplementary Table S1 and **Figure 4A**) had as members several genes encoding pathogenesis related proteins. These included *PR-5, PR-10, PR-10.1, Protein P21*, and seven *chitinases*, four of which were identified by Hawkins et al. (2015) as associated with host resistance to *A. flavus* (Supplementary Table S1). Also in this cluster were several biotic and abiotic stress related genes, including six *GSTs*. Overall, there were 183-annotated and 165 unannotated maize genes in Cluster 6 associated with *A. flavus* infection. The greatest number of genes associated with host resistance expressed in response to *F. verticillioides* clustered in 1F (‘F’ denotes *F. verticillioides-*associated cluster; **Figure 4B** and Supplementary Table S1). This cluster included *PR-5, PR-10, PR-10.1, Protein P21*, and four chitinase genes.

Other clusters contained different defense associated genes. Cluster 1A contained the JA biosynthesis genes *OPR3* and *LOX13*, while cluster 11A contained *OPR2, OPR5* and *LOX4* (Supplementary Table S1). *LOX10* and *LOX11* were clustered in Cluster 2A and 12A in response to *A. flavus*, respectively (Supplementary Table S1). In *F. verticillioides* infected kernels, the expression profile of two LOX genes, *LOX* and *LOX4*, clustered in Cluster 2F, while *LOX10* and *LOX13* clustered in Cluster 7F and 9F, respectively (Supplementary Table S1). *OPR2, OPR3* and *OPR5* clustered in Cluster 9F, 10F and 3F, respectively, in response to *F. verticillioides* infection (Supplementary Table S1).

## Discussion

*Aspergillus flavus* and *F. verticillioides* present worldwide health concerns because of their production of the mycotoxins, aflatoxin, and fumonisin, respectively (Wild et al., 2015). Genetic resistance, which has been successful for the control of many maize diseases, has not been achieved for the diseases caused by these two fungi (Warburton and Williams, 2014; Wild et al., 2015). The inability to identify and move resistance genes into agronomically desirable genotypes is a recognized gap in the development of control strategies for these diseases (Wild et al., 2015).

This study sought to characterize the transcriptional response of developing maize seed to infection by *A. flavus* and *F. verticillioides*, fungi capable of different nutrient associations with maize. In agreement with previous histological observations (Dolezal et al., 2013; Shu et al., 2015), *A. flavus* formed a dense mat of mycelium at the interface of the endosperm and scutellum of the embryo. This mat had the appearance of the type of structure referred to as a biofilm in *Aspergillus* species (Wuren et al., 2014; Reichhardt et al., 2015). We found that *F. verticillioides* did not form this mat. Transcriptional profiling alongside with the histological observations allowed quantification of gene transcription during early recognition events, and during the colonization of different maize seed tissue types. Overall, *A. flavus* colonized seeds much more rapidly and induced gene expression changes in maize seed earlier than did *F. verticillioides* (**Figures 1, 2** and Supplementary Table S1). Differential gene expression was observed in kernels inoculated with either fungus at 4 hpi; however, most genes were more highly expressed at later time points; 24–48 hpi for *A. flavus* and 48–72 hpi for *F. verticillioides*. Infection by *A. flavus* also changed expression of a greater number of maize genes than infection by *F. verticillioides.* In both fungi, more genes were down regulated than up regulated at 4 hpi, including *PR-1* and *PR-10* by *A. flavus*. These genes were in clusters 2A and 6A, respectively (Supplementary Table S1). During infection with *F. verticillioides* a similar number of genes were down regulated as were up regulated at 4 and 12 hpi (**Figure 2**). In contrast, infection by *A. flavus* resulted in nearly 3.5 times more down regulated than up regulated genes at these two time points. These observations suggest that one of the early events in infection by these two fungi is suppression of host gene expression, especially by *A. flavus*.

Differences in observed gene expression in maize in response to the two fungi may have been the result of more extensive kernel colonization by *A. flavus* than by *F. verticillioides*, differences in the colonization of specific kernel tissue types, or the differential response of maize kernels to the two fungi. Examination of histological sections and transcripts reads showed more extensive colonization by *A. flavus*. As reported earlier (Shu et al., 2015) and as shown in this study (**Figure 1**), the two differ in their colonization of maize kernels. *F. verticillioides* appears to cause less initial cell damage in the aleurone than *A. flavus* and *A. flavus* produces an extensive fungal mat adjacent to the scutellum that has not be observed for *F. verticillioides*. The scutellum is a metabolically active tissue known to secrete inhibitory compounds, including PRms (Guo et al., 1999; Murillo et al., 1999). In their studies following timing of PRms expression by *in situ* hybridization, Shu et al. (2015) observed *PRms* transcripts in cells of the aleurone of kernels infected by either *A. flavus* or *F. verticillioides* by 48 hpi, but not in the scutellum of *A. flavus* infected kernels until 72 hpi. Differences in colonization patterns and their adaptation of different strategies for nutrient acquisition could account for dissimilarities in the transcriptional response to the two fungi. Targeted gene expression to change either the type or quantity of fungal inhibitory compounds in the scutellum, for example, could reduce infection by these fungi. Strategies targeted to this tissue may be particularly important for control of aflatoxin contamination as *A. flavus* preferentially colonizes germ tissues and produces more aflatoxin in this tissue than other tissues of the kernel (Smart et al., 1990; Keller et al., 1994). In contrast, *F. verticillioides* produces more fumonisin in the endosperm than in the germ (Bluhm et al., 2008).

The analysis of gene transcripts from infected kernels at five time points, ranging from no visible fungal colonization to extensive colonization, allowed us to determine both temporal and quantitative changes in maize gene expression in response to infection by the two fungi. With this information, we were able to cluster genes of unknown function, which could include putative resistance genes, with characterized defense related genes. Several genes in maize have been associated with resistance to these two fungi by transcriptional profiling studies (Christensen et al., 2014; Dolezal et al., 2014; Lanubile et al., 2014, 2015; Warburton and Williams, 2014; Hawkins et al., 2015; Shu et al., 2015), genome-wide association analysis (Tang et al., 2015), molecular and proteomic analysis (Murillo et al., 1999; Baker et al., 2009a,b), or marker assisted selection (Zhang et al., 2016) (**Table 1**).

**Table 1:** Comparison of maize genes and/or proteins that have been observed in this study and in previous studies.

Gene and/or proteins	Association in previous studies	Association in this study	Reference
PR1	Af and Fv	Af	Lanubile et al., 2010, 2015; Maschietto et al., 2016
PR4	Af and Fv	Af and Fv	Bravo et al., 2003; Dolezal, 2010; Luo et al., 2011
PR5	Af and Fv	Af and Fv	Luo et al., 2011; Lanubile et al., 2013, 2015; Maschietto et al., 2016
PR10	Af and Fv	Af and Fv	Chen et al., 2006, 2010; Dolezal, 2010; Xie et al., 2010; Lanubile et al., 2014
PR10.1	Af	Af and Fv	Xie et al., 2010
Chitinases	Af and Fv	Af and Fv	Cordero et al., 1993; Wu et al., 1994a,b; Ji et al., 2000; Moore et al., 2004; Dolezal, 2010; Lanubile et al., 2010; Peethambaran et al., 2010; Luo et al., 2011; Campos-Bermudez et al., 2013; Hawkins et al., 2015
Thaumatin-like proteins	Af and Fv	Af and Fv	Lanubile et al., 2010, 2014; Luo et al., 2011
β-glucosidase	Af and Fv	Af and Fv	Luo et al., 2011; Campos-Bermudez et al., 2013; Lanubile et al., 2013
CC-NBS-LRR	Af	Af and Fv	Jiang et al., 2011
LRR-RLK	Af and Fv		Luo et al., 2011; Lanubile et al., 2014
WRKYs	Af and Fv	Af and Fv	Lanubile et al., 2010, 2013, 2014; Luo et al., 2011; Campos-Bermudez et al., 2013; Fountain et al., 2013, 2015
MYBs	Af and Fv	Af and Fv	Dolezal, 2010; Lanubile et al., 2010, 2013; Campos-Bermudez et al., 2013
LOXs	Af and Fv	Af and Fv	Wilson et al., 2001; Gao et al., 2008, 2009; Lanubile et al., 2013; Christensen et al., 2014; Dolezal et al., 2014; Scarpari et al., 2014; Maschietto et al., 2015
OPRs	Af and Fv	Af and Fv	Zhang et al., 2005; Christensen et al., 2014; Dolezal et al., 2014
GSTs	Af and Fv	Af and Fv	Dolezal, 2010; Lanubile et al., 2010, 2013; Luo et al., 2011; Campos-Bermudez et al., 2013
Peroxidases	Af and Fv	Af and Fv	Chen et al., 2007; Luo et al., 2011; Dowd and Johnson, 2016
GLBs	Af and Fv	Af and Fv	Chen et al., 2001, 2002, 2007; Lanubile et al., 2010
LEAs	Af	Af and Fv	Chen et al., 2002, 2007; Luo et al., 2011
Zein	Af and Fv	Af and Fv	Lanubile et al., 2010, 2013; Dolezal et al., 2014
HSPs	Af and Fv	Af and Fv	Chen et al., 2002, 2007; Lanubile et al., 2010, 2013; Luo et al., 2011; Campos-Bermudez et al., 2013; Asters et al., 2014
Trypsin inhibitors	Af and Fv	Af and Fv	Chen et al., 1998, 1999a,b, 2007; Tubajika and Damann, 2001; Baker et al., 2009b; Lanubile et al., 2010
Lectin-like protein	Af	Af and Fv	Baker et al., 2009a
Protein kinases	Af and Fv	Af and Fv	Chen et al., 2005; Lanubile et al., 2010, 2013; Luo et al., 2011
Protein phosphatases	Fv	Af and Fv	Lanubile et al., 2010, 2013

*Af, associated with defense response to *Aspergillus flavus*; Fv, associated with defense response to *Fusarium verticillioides*.*

Pathogenesis related proteins, which are often expressed early during infection, are associated with disease resistance of several host species including maize. *PR-4* transcripts are expressed in maize plants upon infection by *F. verticillioides* and *Ustilago maydis* (Bravo et al., 2003; Doehlemann et al., 2008). Maize *PR-5* expression is associated with defense responses to *F. proliferatum, F. subglutinans*, and *A. flavus* (Lanubile et al., 2015). *PR-10*, which has been associated with resistance to *A. flavus* (Chen et al., 2010), was expressed 24 hpi after inoculation. In maize plants, *chitinases* and β*-1, 3- glucanases* were reported to be involved in resistance to *A. flavus* and *F. verticillioides* (Cordero et al., 1993; Lozovaya et al., 1998; Ji et al., 2000; Moore et al., 2004; Warburton and Williams, 2014; Hawkins et al., 2015).

Other defense related proteins associated with resistance include plant chitinases and endoglucanases, which may have cell wall-degrading activities (Huynh et al., 1992; Wu et al., 1994a,b; Bravo et al., 2003), that function downstream of SA and JA/ET signaling pathways (Vidal et al., 1998). Sánchez-Rangel et al. (2012) found that Fumonisin B1 targeted beta-1, 3-glucanase of the germinating maize embryo. Holmes et al. (2008) also identified the maize seed chitinase A and thaumatin-like proteins as inhibitors of *A. flavus* fungal growth and aflatoxin biosynthesis. We observed transcriptional changes of *thaumatin-like proteins* after *A. flavus* or *F. verticillioides* inoculation (Supplementary Table S1). Members of the thaumatin-like proteins were characterized to be PR proteins in *A. thaliana*, barley, wheat and apple (Hejgaard et al., 1991; Hu and Reddy, 1997; Krebitz et al., 2003; Wang et al., 2010). The *PR*-*like* genes that we identified in this study, including the *thaumatin-like proteins*, are putative *PR* genes that might play important roles in maize resistance to a broad spectrum of pathogens.

We also observed elevated expression of several *CC-NBS-LRR* and *LRR-RLK* genes, which are important in the resistance response to pathogens. Over 378 of these *R-like* genes are predicted in the maize genome. One of the 40 members of the *CC-NBS-LRR* genes induced by *A. flavus* and *F. verticillioides* was GRMZM2G032602 (Collins et al., 1998). Elevated expression of this gene has been observed in response to infection by the maize pathogen *Cochliobolus heterostrophus* and by treatment with SA (Cheng et al., 2012), suggesting that the gene is involved in SA-mediated defense responses.

Several studies have shown transcriptional changes in expression of plant genes involved in the biosynthesis and regulation of the plant hormones JA, SA, and ET to be associated with host defense (Nemchenko et al., 2006; Menkir et al., 2008; Pré et al., 2008; Mengiste, 2012; Christensen et al., 2014; Tang et al., 2015). A recent genome-wide association analysis showed the JA biosynthesis pathway to be the most significant metabolic pathway involved in resistance to aflatoxin accumulation (Tang et al., 2015). Christensen et al. (2014) also found JA to be the major defense hormone against *F. verticillioides*, with *LOX12, OPR7*, and *OPR8* playing key roles in this interaction. In our study we observed different *LOX* and *OPR* genes to be differentially expressed in response to *A. flavus* and *F. verticillioides* in maize kernels (Supplementary Table S1).

In some cases, it may be difficult to associate changes in expression of specific hormone biosynthetic genes with host resistance due to cross talk among the JA-, SA-, and ET-mediated pathways (Derksen et al., 2013). For example, *NPR1* (*non-expresser of PR genes 1*), a positive regulator of SA biosynthesis, is also a negative regulator of JA biosynthesis (Mengiste, 2012), We did not find this gene differently expressed in our study even though some SA biosynthesis genes were more highly expressed during infection by *A. flavus.* We also observed infection by either fungus to elevate expression of members of the *MYB* and *ERF* families, which act downstream of the of the JA/ET signaling pathways (Supplementary Table S1). Additionally, we found elevated expression of *PR-5* and several *WRKYs* suggesting that the SA signaling pathway may be involved in maize defense to either fungus. Previous studies showed that the maize *ethylene responsive factor 1* (*ERF1*), a key transcription factor involved in ET and JA signaling, was more highly expressed in the resistant maize inbred TZAR101 than the susceptible B73 in response to *A. flavus* infection (Menkir et al., 2008; Fountain et al., 2013). We also found *AP2-EREBP*, a gene known to integrate ET and JA signaling pathways, and to regulate defense-related gene expression in *Arabidopsis thaliana* (Pré et al., 2008) to be down regulated in response to infection by these two fungi prior to 72 hpi (Supplementary Table S1). In this study, we observed *LOX4, LOX10, LOX11*, and *LOX13* to be differentially expressed in response to both fungi. Nemchenko et al. (2006) found that maize *LOX10* was induced by both biotic and abiotic stresses, such as wounding, cold, *Cochliobolus carbonum*, JA, SA, and ABA treatment. But they reported that *LOX11* was induced only by ABA.

Other phytohormone signaling pathways, such as the ABA, auxin, CKs, BRs, and GA pathways, interact with SA, JA and ET signaling pathways and form a regulatory network which is important in defense response to external stimulus (Angra-Sharma and Sharma, 1999; Siemens et al., 2006; Walters and McRoberts, 2006; Pieterse et al., 2009; Zhang et al., 2010; Iglesias et al., 2011; Behr et al., 2012; Denancé et al., 2013; Derksen et al., 2013; Yang et al., 2013; Zhu et al., 2015). We detected transcriptional changes of genes that are associated with ABA, auxin, and GA pathways that affect the three innate defense pathways (Supplementary Table S1), suggesting a complex regulatory network triggered by these fungi. Our results indicate that upregulation of ABA/auxin signaling pathways may contribute to the inhibition of SA signaling. The inhibition of SA signaling would attenuate host resistance and promote fungal colonization. ABA and auxin signaling pathways suppress SA signaling in plants (Wang et al., 2007; Pieterse et al., 2009). Elevated auxin levels and transcriptional induction of auxin-responsive genes were also observed in maize during *U. maydis* infection (Turian and Hamilton, 1960; Doehlemann et al., 2008). Our data indicate that both GA signaling and GA degradation pathways changed during infection.

Downregulation of the GA signaling pathway may release the inhibition of JA/ET signaling in the kernel. In *A. thaliana*, GA produced by *Gibberella fujikuroi* inhibits JA-dependent necrotroph resistance via GA-mediated degradation of DELLA proteins (Navarro et al., 2008). The role of GA in maize kernels may be more complex as it is also involved in seed development by stimulating the production of proteases and α-amylases necessary for the hydrolysis of endosperm starch and proteins, a process that ABA inhibits (Harvey and Oaks, 1974). We also observed elevated expression of BR and CK signaling pathways in response to infection by either fungus. BRs have been shown to contribute to disease resistance in *A. thaliana*, tobacco and rice plants (Nakashita et al., 2003; Belkhadir et al., 2012). Collectively, our data support the conclusion that an interacting hormone network contributes to maize resistance to *A. flavus* and *F. verticillioides* in developing maize kernels.

Several studies have shown that glutathiones produced by GSTs to be important in resistance to abiotic and biotic stress through their action as soluble antioxidants (Mauch and Dudler, 1993; Ogawa, 2005; Sytykiewicz, 2011; Wisser et al., 2011). As an example, elevated GST levels were found during infection of wheat by *Erysiphe graminis* (Dudler et al., 1991) and *A. thaliana* by *Alternaria brassicicola* (Mukherjee et al., 2010). We also observed elevated transcription of GST genes during infection by either *A. flavus* or *F. verticillioides*, suggesting a role of glutathiones in this host–pathogen interaction.

In this study, we directly compared transcriptional changes in maize seeds during colonization by the two major mycotoxin-producing fungi on maize. By pairing histological and transcriptional examinations during disease development in the field, we were able to associate transcriptional changes with tissue colonization by the two fungi under conditions conducive for their growth. The study revealed similarities and differences in the response of maize to two fungi differing in tropic relationships with the host. We were able to construct a comprehensive gene expression database for those interested in further characterization of these two diseases and those interested in breeding for resistance.

Using hierarchal clustering analysis, we were able to place genes into 12 clusters representing genes with similar expression profiles (Supplementary Table S1 and **Figure 4**). The placement of genes in clusters based on the timing and magnitude of their expression along with genes of unknown function promises to aid in future understanding of maize resistance regulatory circuits, as well as guide the selection of candidate genes for further studies on their role in host resistance. While expression of known defense related genes has been associated with host resistance to these two diseases in maize, their expression alone has not been shown to be sufficient for adequate resistance. This is presumably due to lack of sufficient expression of these genes or to improper timing of expression of downstream regulatory genes. The gene profile analysis provided in this manuscript will serve as a genetic resource and aid in the identification of candidate resistance genes.

## Author Contributions

XS and CW designed the experiments. XS and DL performed the experimental work. XS performed the data analysis. GP supervised the research and manuscript preparation. All authors wrote and edited the manuscript.

## Conflict of Interest Statement

The authors declare that the research was conducted in the absence of any commercial or financial relationships that could be construed as a potential conflict of interest.

## Footnotes

1 https://figshare.com/projects/Comparative_histological_and_transcriptional_analysis_of_maize_kernels_infected_with_Aspergillus_flavus_and_Fusarium_verticillioides/26473

## References

[B1] Angra-Sharma R. Sharma D. K. (1999). Cytokinins in pathogenesis and disease resistance of *Pyrenophora teres*-barley and *Dreschslera maydis*-maize interactions during early stages of infection. *Mycopathologia* 148 87–95. 10.1023/A:1007126025955 11189749

[B2] Asters M. C. Williams W. P. Perkins A. D. Mylroie J. E. Windham G. L. Shan X. (2014). Relating significance and relations of differentially expressed genes in response to *Aspergillus flavus* infection in maize. *Sci. Rep.* 4:4815. 10.1038/srep04815 24770700 PMC4001098

[B3] Bacon C. W. Bennett R. M. Hinton D. M. Voss K. A. (1992). Scanning electron microscopy of *Fusarium moniliforme* within asymptomatic corn kernels and kernels associated with equine leukoencephalomalacia. *Plant Dis.* 76 144–148. 10.1094/PD-76-0144

[B4] Baker R. L. Brown R. L. Chen Z. Y. Cleveland T. E. Fakhoury A. M. (2009a). A maize lectin-like protein with antifungal activity against *Aspergillus flavus*. *J. Food Prot.* 72 120–127. 19205472 10.4315/0362-028x-72.1.120

[B5] Baker R. L. Brown R. L. Chen Z. Y. Cleveland T. E. Fakhoury A. M. (2009b). A maize trypsin inhibitor (ZmTIp) with limited activity against *Aspergillus flavus*. *J. Food Prot.* 72 185–188. 19205484 10.4315/0362-028x-72.1.185

[B6] Behr M. Motyka V. Weihmann F. Malbeck J. Deising H. B. Wirsel S. G. (2012). Remodeling of cytokinin metabolism at infection sites of *Colletotrichum graminicola* on maize leaves. *Mol. Plant Microbe Interact.* 25 1073–1082. 10.1094/MPMI-01-12-0012-R 22746825

[B7] Belkhadir Y. Jaillais Y. Epple P. Balsemão-Pires E. Dangl J. L. Chory J. (2012). Brassinosteroids modulate the efficiency of plant immune responses to microbe-associated molecular patterns. *Proc. Natl. Acad. Sci. U.S.A.* 109 297–302. 10.1073/pnas.1112840108 22087001 PMC3252953

[B8] Bluhm B. H. Kim H. Butchko R. A. E. Woloshuk C. P. (2008). Involvement of ZFR1 of *Fusarium verticillioides* in kernel colonization and the regulation of FST1 a putative sugar transporter gene required for fumonisin biosynthesis on maize kernels. *Mol. Plant Pathol.* 9 203–211. 10.1111/j.1364-3703.2007.00458.x 18705852 PMC6640386

[B9] Bravo J. M. Campo S. Murillo I. Coca M. San Segundo B. (2003). Fungus- and wound-induced accumulation of mRNA containing a class II chitinase of the pathogenesis-related protein 4 (PR-4) family of maize. *Plant Mol. Biol.* 52 745–759. 10.1023/A:1025016416951 13677464

[B10] Brown R. L. Menkir A. Chen Z. Y. Bhatnagar D. Yu J. Yao H. (2013). Breeding aflatoxin-resistant maize lines using recent advances in technologies - a review. *Food Addit. Contam. Part A Chem. Anal. Control Expo. Risk Assess.* 30 1382–1391. 10.1080/19440049.2013.812808 23859902

[B11] Campos-Bermudez V. A. Fauguel C. M. Tronconi M. A. Casati P. Presello D. A. Andreo C. S. (2013). Transcriptional and metabolic changes associated to the infection by *Fusarium verticillioides* in maize inbreds with contrasting ear rot resistance. *PLOS ONE* 8:e61580. 10.1371/journal.pone.0061580 23637860 PMC3630110

[B12] Caspi R. Altman T. Dreher K. Fulcher C. A. Subhraveti P. Keseler I. M. (2012). The MetaCyc database of metabolic pathways and enzymes and the BioCyc collection of pathway/genome databases. *Nucleic Acids Res.* 40 D742–D753. 10.1093/nar/gkv1164 22102576 PMC3245006

[B13] Castellana N. E. Shen Z. He Y. Walley J. W. Cassidy C. J. Briggs S. P. (2013). An automated proteogenomic method utilizes mass spectrometry to reveal novel genes in *Zea mays*. *Mol. Cell. Proteomics* 13 157–167. 10.1074/mcp.M113.031260 24142994 PMC3879611

[B14] Chen Z. Y. Brown R. L. Cleveland T. E. Damann K. E. Russin J. S. (2001). Comparison of constitutive and inducible maize kernel proteins of genotypes resistant or susceptible to aflatoxin production. *J. Food Prot.* 64 1785–1792. 10.4315/0362-028X-64.11.1785 11726160

[B15] Chen Z. Y. Brown R. L. Damann K. E. Cleveland T. E. (2002). Identification of unique or elevated levels of kernel proteins in aflatoxin-resistant maize genotypes through proteome analysis. *Phytopathology* 92 1084–1094. 10.1094/PHYTO.2002.92.10.1084 18944219

[B16] Chen Z. Y. Brown R. L. Damann K. E. Cleveland T. E. (2007). Identification of maize kernel endosperm proteins associated with resistance to aflatoxin contamination by *Aspergillus flavus*. *Phytopathology* 97 1094–1103. 10.1094/PHYTO-97-9-1094 18944174

[B17] Chen Z. Y. Brown R. L. Damann K. E. Cleveland T. E. (2010). PR10 expression in maize and its effect on host resistance against *Aspergillus flavus* infection and aflatoxin production. *Mol. Plant Pathol.* 11 69–81. 10.1111/j.1364-3703.2009.00574.x 20078777 PMC6640484

[B18] Chen Z. Y. Brown R. L. Lax A. R. Cleveland T. E. Russin J. S. (1999a). Inhibition of plant-pathogenic fungi by a corn trypsin inhibitor overexpressed in *Escherichia coli*. *Appl. Environ. Microbiol.* 65 1320–1324. 10049901 10.1128/aem.65.3.1320-1324.1999 PMC91182

[B19] Chen Z. Y. Brown R. L. Lax A. R. Guo B. Z. Cleveland T. E. Russin J. S. (1998). Resistance to *Aspergillus flavus* in corn kernels is associated with a 14-kDa protein. *Phytopathology* 88 276–281. 10.1094/PHYTO.1998.88.4.276 18944949

[B20] Chen Z. Y. Brown R. L. Menkir A. Damann K. Cleveland T. E. (2005). Proteome analysis of near isogenic maize lines differing in the level of resistance against *Aspergillus flavus* infection/aflatoxin production. *Phytopathology* 95:S19.

[B21] Chen Z. Y. Brown R. L. Rajasekaran K. Damann K. E. Cleveland T. E. (2006). Identification of a maize kernel pathogenesis-related protein and evidence for its involvement in resistance to *Aspergillus flavus* infection and aflatoxin production. *Phytopathology* 96 87–95. 10.1094/PHYTO-96-0087 18944208

[B22] Chen Z. Y. Brown R. L. Russin J. S. Lax A. R. Cleveland T. E. (1999b). A corn trypsin inhibitor with antifungal activity inhibits *Aspergillus flavus* alpha-amylase. *Phytopathology* 89 902–907. 10.1094/PHYTO.1999.89.10.902 18944733

[B23] Cheng Y. Li X. Jiang H. Ma W. Miao W. Yamada T. (2012). Systematic analysis and comparison of nucleotide-binding site disease resistance genes in maize. *FEBS J.* 279 2431–2443. 10.1111/j.1742-4658.2012.08621.x 22564701

[B24] Christensen S. A. Nemchenko A. Park Y. S. Borrego E. Huang P. C. Schmelz E. A. (2014). The novel monocot-specific 9-lipoxygenase ZmLOX12 is required to mount an effective jasmonate-mediated defense against *Fusarium verticillioides* in maize. *Mol. Plant Microbe Interact.* 27 1263–1276. 10.1094/MPMI-06-13-0184-R 25122482

[B25] Collins N. C. Webb C. A. Seah S. Ellis J. G. Hulbert S. H. Pryor A. (1998). The isolation and mapping of disease resistance gene analogs in maize. *Mol. Plant Microbe Interact.* 11 968–978. 10.1094/MPMI.1998.11.10.968 9768514

[B26] Cordero M. J. Raventos D. San Segundo B. (1993). Differential expression and induction of chitinases and β-13-glucanases in response to fungal infection during germination of maize seeds. *Mol. Plant Microbe Interact.* 7 23–31. 10.1094/MPMI-7-0023

[B27] Denancé N. Sánchez-Vallet A. Goffner D. Molina A. (2013). Disease resistance or growth: the role of plant hormones in balancing immune responses and fitness costs. *Front. Plant Sci.* 4:155. 10.3389/fpls.2013.00155 23745126 PMC3662895

[B28] Derksen H. Rampitsch C. Daayf F. (2013). Signaling cross-talk in plant disease resistance. *Plant Sci.* 207 79–87. 10.1016/j.plantsci.2013.03.004 23602102

[B29] Doehlemann G. Wahl R. Horst R. J. Voll L. M. Usadel B. Poree F. (2008). Reprogramming a maize plant: transcriptional and metabolic changes induced by the fungal biotroph *Ustilago maydis*. *Plant J.* 56 181–195. 10.1111/j.1365-313X.2008.03590.x 18564380

[B30] Dolezal A. L. (2010). *Interactions between Aspergillus flavus and the Developing Maize Kernel.* Doctoral dissertation, North Carolina State University Raleigh, NC.

[B31] Dolezal A. L. Obrian G. R. Nielsen D. M. Woloshuk C. P. Boston R. S. Payne G. A. (2013). Localization, morphology and transcriptional profile of *Aspergillus flavus* during seed colonization. *Mol. Plant Pathol.* 14 898–909. 10.1111/mpp.12056 23834374 PMC6638638

[B32] Dolezal A. L. Shu X. OBrian G. R. Nielsen D. M. Woloshuk C. P. Boston R. S. (2014). *Aspergillus flavus* infection induces transcriptional and physical changes in developing maize kernels. *Front. Microbiol.* 5:384. 10.3389/fmicb.2014.00384 25132833 PMC4117183

[B33] Dowd P. F. Johnson E. T. (2016). Maize peroxidase Px5 has a highly conserved sequence in inbreds fungal resistant to mycotoxin producing fungi which enhances resistance insect. *J. Plant Res.* 129 13–20. 10.1007/s10265-015-0770-3 26659597

[B34] Dudler R. Hertig C. Rebmann G. Bull J. Mauch F. (1991). A pathogen-induced wheat gene encodes a protein homologous to glutathione-S-transferases. *Mol. Plant Microbe Interact.* 4 14–18. 10.1094/MPMI-4-014 1799693

[B35] Duncan K. E. Howard R. J. (2010). Biology of maize kernel infection by *Fusarium verticillioides*. *Mol. Plant Microbe Interact.* 23 6–16. 10.1094/MPMI-23-1-0006 19958134

[B36] Fountain J. C. Raruang Y. Luo M. Brown R. L. Chen Z. Y. (2013). Maize WRKY transcription factors and their potential roles in regulating defense gene expression during *Aspergillus flavus* infection. *Phytopathology* 103(Suppl. 1):S1.4.

[B37] Fountain J. C. Raruang Y. Luo M. Brown R. L. Guo B. Chen Z.-Y. (2015). Potential roles of WRKY transcription factors in regulating host defense responses during *Aspergillus flavus* infection of immature maize kernels. *Physiol. Mol. Plant Pathol.* 89 31–40. 10.1016/j.pmpp.2014.11.005

[B38] Gao X. Brodhagen M. Isakeit T. Brown S. H. Gobel C. Betran J. (2009). Inactivation of the lipoxygenase ZmLOX3 increases susceptibility of maize to *Aspergillus* spp. *Mol. Plant Microbe Interact.* 22 222–231. 10.1094/MPMI-22-2-0222 19132874 PMC4545248

[B39] Gao X. Stumpe M. Feussner I. Kolomiets M. (2008). A novel plastidial lipoxygenase of maize (*Zea mays*) ZmLOX6 encodes for a fatty acid hydroperoxide lyase and is uniquely regulated by phytohormones and pathogen infection. *Planta* 227 491–503. 10.1007/s00425-007-0634-8 17922288

[B40] Guo B. Z. Cleveland T. E. Brown R. L. Widstrom N. W. Lynch R. E. Russin J. S. (1999). Distribution of antifungal proteins in maize kernel tissues using immunochemistry. *J. Food Prot.* 62 295–299. 10.4315/0362-028X-62.3.295 10090253

[B41] Harvey B. M. R. Oaks A. (1974). The role of gibberellic acid in the hydrolysis of endosperm reserves in *Zea mays*. *Planta* 121 67–74. 10.1007/BF00384007 24442735

[B42] Hawkins L. K. Mylroie J. E. Oliveira D. A. Smith J. S. Ozkan S. Windham G. L. (2015). Characterization of the maize chitinase genes and their effect on *Aspergillus flavus* and aflatoxin accumulation resistance. *PLOS ONE* 10:e0126185. 10.1371/journal.pone.0126185 26090679 PMC4475072

[B43] Hejgaard J. Jacobsen S. Svendsen I. (1991). Two antifungal thaumatin-like proteins from barley grain. *FEBS Lett.* 291 127–131. 10.1016/0014-5793(91)81119-S 1936240

[B44] Holmes R. A. Boston R. S. Payne G. A. (2008). Diverse inhibitors of aflatoxin biosynthesis. *Appl. Microbiol. Biotechnol.* 78 559–572. 10.1007/s00253-008-1362-0 18246345

[B45] Hu X. Reddy A. S. (1997). Cloning and expression of a PR5-like protein from *Arabidopsis*: inhibition of fungal growth by bacterially expressed protein. *Plant Mol. Biol.* 34 949–959. 10.1023/A:1005893119263 9290646

[B46] Huynh Q. K. Hironaka C. M. Levine E. B. Smith C. E. Borgmeyer J. R. Shah D. M. (1992). Antifungal proteins from plants. Purification, molecular cloning, and antifungal properties of chitinases from maize seed. *J. Biol. Chem.* 267 6635–6640. 1551872

[B47] Iglesias M. J. Terrile M. C. Casalongue C. A. (2011). Auxin and salicylic acid signalings counteract the regulation of adaptive responses to stress. *Plant Signal. Behav.* 6 452–454. 10.4161/psb.6.3.14676 21358272 PMC3142437

[B48] Ji C. Norton R. A. Wicklow D. T. Dowd P. F. (2000). Isoform patterns of chitinase and beta-1,3-glucanase in maturing corn kernels (*Zea mays* L.) associated with *Aspergillus flavus* milk stage infection. *J. Agric. Food Chem.* 48 507–511. 10.1021/jf9905119 10691666

[B49] Jiang T. Zhou B. Luo M. Abbas H. K. Kemerait R. Lee R. D. (2011). Expression analysis of stress-related genes in kernels of different maize (*Zea mays* L.) inbred lines with different resistance to aflatoxin contamination. *Toxins* 3 538–550. 10.3390/toxins3060538 22069724 PMC3202848

[B50] Keller N. P. Butchko R. Sarr B. Phillips T. D. (1994). A visual pattern of mycotoxin production in maize kernels by *Aspergillus* spp. *Phytopathology* 84 483–488. 10.1094/Phyto-84-483

[B51] Kelley R. Y. Williams W. P. Mylroie J. E. Boykin D. L. Harper J. W. Windham G. L. (2012). Identification of maize genes associated with host plant resistance or susceptibility to *Aspergillus flavus* infection and aflatoxin accumulation. *PLOS ONE* 7:e36892. 10.1371/journal.pone.0036892 22606305 PMC3351445

[B52] Kim M. Zhang H. Woloshuk C. Shim W. B. Yoon B. J. (2015). Computational identification of genetic subnetwork modules associated with maize defense response to *Fusarium verticillioides*. *BMC Bioinformatics* 13:S12. 10.1186/1471-2105-16-S13-S12 26423221 PMC4597171

[B53] Koehler B. (1942). Natural mode of entrance of fungi into corn ears and some symptoms that indicate infection. *J. Agric. Res.* 64 421–442.

[B54] Krebitz M. Wagner B. Ferreira F. Peterbauer C. Campillo N. Witty M. (2003). Plant-based heterologous expression of Mal d 2, a thaumatin-like protein and allergen of apple (*Malus domestica*), and its characterization as an antifungal protein. *J. Mol. Biol.* 329 721–730. 10.1016/S0022-2836(03)00403-0 12787673

[B55] Lanubile A. Bernardi J. Marocco A. Logrieco A. Paciolla C. (2012). Differential activation of defense genes and enzymes in maize genotypes with contrasting levels of resistance to *Fusarium verticillioides*. *Environ. Exp. Bot.* 78 39–46. 10.1016/j.envexpbot.2011.12.006

[B56] Lanubile A. Ferrarini A. Maschietto V. Delledonne M. Marocco A. Bellin D. (2014). Functional genomic analysis of constitutive and inducible defense responses to *Fusarium verticillioides* infection in maize genotypes with contrasting ear rot resistance. *BMC Genomics* 15:710. 10.1186/1471-2164-15-710 25155950 PMC4153945

[B57] Lanubile A. Logrieco A. Battilani P. Proctor R. H. Marocco A. (2013). Transcriptional changes in developing maize kernels in response to fumonisin-producing and nonproducing strains of *Fusarium verticillioides*. *Plant Sci.* 210 183–192. 10.1016/j.plantsci.2013.05.020 23849125

[B58] Lanubile A. Maschietto V. De Leonardis S. Battilani P. Paciolla C. Marocco A. (2015). Defense responses to mycotoxin-producing fungi *Fusarium proliferatum, F. subglutinans*, and *Aspergillus flavus* in kernels of susceptible and resistant maize genotypes. *Mol. Plant Microbe Interact.* 28 546–557. 10.1094/MPMI-09-14-0269-R 26024441

[B59] Lanubile A. Pasini L. Marocco A. (2010). Differential gene expression in kernels and silks of maize lines with contrasting levels of ear rot resistance after *Fusarium verticillioides* infection. *J. Plant Physiol.* 167 1398–1406. 10.1016/j.jplph.2010.05.015 20650545

[B60] Lawrence C. J. Dong Q. Polacco M. L. Seigfried T. E. Brendel V. (2004). MaizeGDB, the community database for maize genetics and genomics. *Nucleic Acids Res.* 32 D393–D397. 10.1093/nar/gkh011 14681441 PMC308746

[B61] Lillehoj E. B. Kwolek W. F. Fennell D. I. Milburn M. S. (1975). Aflatoxin incidence and association with bright greenish-yellow fluorescence and insect damage in a limited survey of freshly harvested high-moisture corn. *Cereal Chem.* 52 403–411.

[B62] Ling Y. Du Z. Zhang Z. Su Z. (2010). ProFITS of maize: a database of protein families involved in the transduction of signaling in the maize genome. *BMC Genomics* 11:580. 10.1186/1471-2164-11-580 20955618 PMC3091727

[B63] Livingston D. P. Henson C. A. Tuong T. D. Wise M. L. Tallury S. P. Duke S. H. (2013). Histological analysis and 3D reconstruction of winter cereal crowns recovering from freezing: a unique response in oat (*Avena sativa* L.). *PLOS ONE* 8:e53468. 10.1371/journal.pone.0053468 23341944 PMC3544926

[B64] Livingston D. P. Tuong T. D. Haigler C. H. Avci U. Tallury S. P. (2009). Rapid microwave processing of winter cereals for histology allows identification of separate zones of freezing injury in the crown. *Crop Sci.* 49 1837–1842. 10.2135/cropsci2009.02.0077

[B65] Lozovaya V. V. Waranyuwat A. Widholm J. M. (1998). Beta-1,3-Glucanase and resistance to *Aspergillus flavus* infection in maize. *Crop Sci.* 38 1255–1260. 10.2135/cropsci1998.0011183X003800050024x

[B66] Luo M. Brown R. L. Chen Z. Y. Menkir A. Yu J. Bhatnagar D. (2011). Transcriptional profiles uncover *Aspergillus flavus*-induced resistance in maize kernels. *Toxins* 3 766–786. 10.3390/toxins3070766 22069739 PMC3202853

[B67] Lyons E. Freeling M. (2008). How to usefully compare homologous plant genes and chromosomes as DNA sequences. *Plant J.* 53 661–673. 10.1111/j.1365-313X.2007.03326.x 18269575

[B68] Marsh S. Payne G. (1984). Preharvest infection of corn silks and kernels by *Aspergillus flavus*. *Phytopathology* 74 1284–1289. 10.1094/Phyto-74-1284

[B69] Maschietto V. Lanubile A. Leonardis S. Marocco A. Paciolla C. (2016). Constitutive expression of pathogenesis-related proteins and antioxydant enzyme activities triggers maize resistance towards *Fusarium verticillioides*. *J. Plant Physiol.* 200 53–61. 10.1016/j.jplph.2016.06.006 27340858

[B70] Maschietto V. Marocco A. Malachova A. Lanubile A. (2015). Resistance to *Fusarium verticillioides* and fumonisin accumulation in maize inbred lines involves an earlier and enhanced expression of lipoxygenase (LOX) genes. *J. Plant Physiol.* 188 9–18. 10.1016/j.jplph.2015.09.003 26398628

[B71] Matasci N. McKay S. (2013). Phylogenetic analysis with the iPlant discovery environment. *Curr. Protoc. Bioinformatics* 42 6.13.1–6.13.13. 23749754 10.1002/0471250953.bi0613s42

[B72] Mauch F. Dudler R. (1993). Differential induction of distinct glutathione-S-transferases of wheat by xenobiotics and by pathogen attack. *Plant Physiol.* 102 1193–1201. 10.1104/pp.102.4.1193 8278547 PMC158905

[B73] Mengiste T. (2012). Plant immunity to necrotrophs. *Annu. Rev. Phytopathol.* 50 267–294. 10.1146/annurev-phyto-081211-172955 22726121

[B74] Menkir A. Brown R. L. Bandyopadhyay R. Cleveland T. E. (2008). Registration of six tropical maize germplasm lines with resistance to aflatoxin contamination. *J. Plant Regist.* 2 246–250. 10.3198/jpr2008.01.0028crg

[B75] Moore K. G. Price M. S. Boston R. S. Weissinger A. K. Payne G. A. (2004). A chitinase from Tex6 maize kernels inhibits growth of *Aspergillus flavus*. *Phytopathology* 94 82–87. 10.1094/PHYTO.2004.94.1.82 18943823

[B76] Mukherjee A. K. Carpm M. J. Zuchman R. Ziv T. Horwitz B. A. Gepstein S. (2010). Proteomics of the response of *Arabidopsis thaliana* to infection with *Alternaria brassicicola*. *J. Proteomics* 73 709–720. 10.1016/j.jprot.2009.10.005 19857612

[B77] Munkvold G. P. (2003). Cultural and genetic approaches managing mycotoxins in maize. *Annu. Rev. Phytopathol.* 41 99–116. 10.1146/annurev.phyto.41.052002.095510 12730397

[B78] Munkvold G. P. McGee D. C. Carlton W. M. (1997). Importance of different pathways for maize kernel infection by *Fusarium moniliforme*. *Phytopathology* 87 209–217. 10.1094/PHYTO.1997.87.2.209 18945144

[B79] Murillo I. Cavallarin L. Segundo B. S. (1999). Cytology of infection of maize seedlings by *Fusarium moniliforme* and immunolocalization of the pathogenesis-related PRms protein. *Phytopathology* 89 737–747. 10.1094/PHYTO.1999.89.9.737 18944701

[B80] Musungu B. M. Bhatnagar D. Brown R. L. Payne G. A. OBrian G. Fakhoury A. M. (2016). A network approach of gene co-expression in the *Zea mays*/*Aspergillus flavus* pathosystem to map host/pathogen interaction pathways. *Front. Genet.* 7:206. 10.3389/fgene.2016.00206 27917194 PMC5116468

[B81] Nakashita H. Yasuda M. Nitta T. Asami T. Fujioka S. Arai Y. (2003). Brassinosteroid functions in a broad range of disease resistance in tobacco and rice. *Plant J.* 33 887–898. 10.1046/j.1365-313X.2003.01675.x 12609030

[B82] Navarro L. Bari R. Achard P. Lisón P. Nemri A. Harberd N. P. (2008). DELLAs control plant immune responses by modulating the balance of jasmonic acid and salicylic acid signaling. *Curr. Biol.* 18 650–655. 10.1016/j.cub.2008.03.060 18450451

[B83] Nemchenko A. Kunze S. Feussner I. Kolomiets M. (2006). Duplicate maize 13-lipoxygenase genes are differentially regulated by circadian rhythm, cold stress, wounding, pathogen infection, and hormonal treatments. *J. Exp. Bot.* 57 3767–3779. 10.1093/jxb/erl137 17005920

[B84] Ogawa K. (2005). Glutathione-associated regulation of plant growth and stress responses. *Antioxid. Redox Signal.* 7 973–981. 10.1089/ars.2005.7.973 15998252

[B85] Payne A. G. Yu J. (2010). “Ecology, development and gene regulation in *Aspergillus flavus*,” in *Aspergillus: Molecular Biology and Genomics* eds Machida M. Gomi K. (Norfolk, VA: Caister Academic Press) 157–171.

[B86] Payne G. A. (1992). Aflatoxin in maize. *Crit. Rev. Plant Sci.* 10 423–440. 10.1080/07352689209382320

[B87] Payne G. A. Cassel D. K. Adkins C. R. (1986). Reduction of aflatoxin contamination in corn by irrigation and tillage. *Phytopathology* 76 679–684. 10.1094/Phyto-76-679

[B88] Pechanova O. Pechan T. Williams W. P. Luthe D. S. (2011). Proteomic analysis of the maize rachis: potential roles of constitutive and induced proteins in resistance to *Aspergillus flavus* infection and aflatoxin accumulation. *Proteomics* 11 114–127. 10.1002/pmic.201000368 21182199

[B89] Peethambaran B. Hawkins L. Windham G. L. Williams W. P. Luthe D. S. (2010). Anti-fungal activity of maize silk proteins and role of chitinases in *Aspergillus flavus* resistance. *Toxin Rev.* 29 27–39. 10.3109/15569540903402874

[B90] Pei-Bao Z. Ren A. Z. Xu H. J. Li D. C. (2010). The gene fpk1 encoding a cAMP-dependent protein kinase catalytic subunit homolog, is required for hyphal growth, spore germination, and plant infection in *Fusarium verticillioides*. *J. Microbiol. Biotechnol.* 20 208–216. 20134254

[B91] Pieterse C. M. Leon-Reyes A. Van der Ent S. Van Wees S. C. (2009). Networking by small-molecule hormones in plant immunity. *Nat. Chem. Biol.* 5 308–316. 10.1038/nchembio.164 19377457

[B92] Pré M. Atallah M. Champion A. De Vos M. Pieterse C. M. Memelink J. (2008). The AP2/ERF domain transcription factor ORA59 integrates jasmonic acid and ethylene signals in plant defense. *Plant Physiol.* 147 1347–1357. 10.1104/pp.108.117523 18467450 PMC2442530

[B93] Reichhardt C. Ferreira J. A. Joubert L. M. Clemons K. V. Stevens D. A. Cegelski L. (2015). Analysis of the *Aspergillus fumigatus* biofilm extracellular matrix by solid-state nuclear magnetic resonance spectroscopy. *Eukaryot. Cell* 14 1064–1072. 10.1128/EC.00050-15 26163318 PMC4621319

[B94] Sánchez-Rangel D. Sánchez-Nieto S. Plasencia J. (2012). Fumonisin B1, a toxin produced by *Fusarium verticillioides*, modulates maize beta-1,3-glucanase activities involved in defense response. *Planta* 235 965–978. 10.1007/s00425-011-1555-0 22120123

[B95] Scarpari M. Punelli M. Scala V. Zaccaria M. Nobili C. Ludovici M. (2014). Lipids in *Aspergillus flavus*-maize interaction. *Front. Microbiol.* 5:74. 10.3389/fmicb.2014.00074 24578700 PMC3936598

[B96] Scheidegger K. A. Payne G. A. (2003). Unlocking the secrets behind secondary metabolism: a review of *Aspergillus flavus* from pathogenicity to functional genomics. *Toxin Rev.* 22 423–459. 10.1081/TXR-120024100

[B97] Schnable P. S. Ware D. Fulton R. S. Stein J. C. Wei F. Pasternak S. (2009). The B73 maize genome: complexity, diversity, and dynamics. *Science* 326 1112–1115. 10.1126/science.1178534 19965430

[B98] Shu X. (2014). *Pathogenesis and Host Response during Infection of Maize Kernels by Aspergillus flavus and Fusarium verticillioides.* Doctoral dissertation, North Carolina State University Raleigh, NC.

[B99] Shu X. Livingston D. P. Franks R. G. Boston R. S. Woloshuk C. P. Payne G. A. (2015). Tissue specific gene expression in maize seeds during colonization by *Aspergillus flavus* and *Fusarium verticillioides*. *Mol. Plant Pathol.* 16 662–674. 10.1111/mpp.12224 25469958 PMC6638326

[B100] Siemens J. Keller I. Sarx J. Kunz S. Schuller A. Nagel W. (2006). Transcriptome analysis of *Arabidopsis* clubroots indicate a key role for cytokinins in disease development. *Mol. Plant Microbe Interact.* 19 480–494. 10.1094/MPMI-19-0480 16673935

[B101] Smart M. G. Wicklow D. T. Caldwell R. W. (1990). Pathogenesis in *Aspergillus* ear rot of maize: light microscopy of fungal spread from wounds. *Phytopathology* 80 1287–1294. 10.1094/Phyto-80-1287

[B102] Sobek E. A. Munkvold G. P. (1999). European corn borer (Lepidoptera: Pyralidae) larvae as vectors of *Fusarium moniliforme*, causing kernel rot and symptomless infection of maize kernels. *J. Econ. Entomol.* 92 503–509. 10.1093/jee/92.3.503

[B103] Song W. Wang B. Li X. Wei J. Chen L. Zhang D. (2015). Identification of immune related LRR-containing genes in maize (*Zea mays* L.) by genome-wide sequence analysis. *Int. J. Genomics* 2015:231358. 10.1155/2015/231358 26609518 PMC4645488

[B104] St. Leger R. J. Screen S. E. Shams-Pirzadeh B. (2000). Lack of host specialization in *Aspergillus flavus*. *Appl. Environ. Microbiol.* 66 320–324. 10.1128/AEM.66.1.320-324.2000 10618242 PMC91824

[B105] Sytykiewicz H. (2011). Expression patterns of glutathione transferase gene (*GstI*) in maize seedlings under juglone-induced oxidative stress. *Int. J. Mol. Sci.* 12 7982–7995. 10.3390/ijms12117982 22174645 PMC3233451

[B106] Tang J. D. Perkins A. Williams W. P. Warburton M. L. (2015). Using genome-wide associations to identify metabolic pathways involved in maize aflatoxin accumulation resistance. *BMC Genomics* 16:673. 10.1186/s12864-015-1874-9 26334534 PMC4558830

[B107] Trapnell C. Roberts A. Goff L. Pertea G. Kim D. Kelley D. R. (2012). Differential gene and transcript expression analysis of RNA-seq experiments with TopHat and Cufflinks. *Nat. Protoc.* 7 562–578. 10.1038/nprot.2012.016 22383036 PMC3334321

[B108] Tubajika K. M. Damann K. E. (2001). Sources of resistance to aflatoxin production in maize. *J. Agric. Food Chem.* 49 2652–2656. 10.1021/jf001333i 11368650

[B109] Turian G. Hamilton R. H. (1960). Chemical detection of 3-indolylacetic acid in *Ustilago zeae* tumors. *Biochim. Biophys. Acta* 41 148–150. 10.1016/0006-3002(60)90381-4 13839903

[B110] Usadel B. Poree F. Nagel A. Lohse M. Czedik-Eysenberg A. Stitt M. (2009). A guide to using MapMan to visualize and compare omics data in plants: a case study in the crop species, Maize. *Plant Cell Environ.* 32 1211–1229. 10.1111/j.1365-3040.2009.01978.x 19389052

[B111] Vidal S. Eriksson A. R. B. Montesano M. Denecke J. Palva E. T. (1998). Cell wall-degrading enzymes from *Erwinia carotovora* cooperate in the salicylic acid-independent induction of a plant defense response. *Mol. Plant Microbe Interact.* 11 23–32. 10.1094/MPMI.1998.11.1.23

[B112] Walters D. R. McRoberts N. (2006). Plants and biotrophs: a pivotal role for cytokinins? *Trends Plant Sci.* 11 581–586. 17092762 10.1016/j.tplants.2006.10.003

[B113] Wang D. Pajerowska-Mukhtar K. Culler A. H. Dong X. (2007). Salicylic acid inhibits pathogen growth in plants through repression of the auxin signaling pathway. *Curr. Biol.* 17 1784–1790. 10.1016/j.cub.2007.09.025 17919906

[B114] Wang X. Tang C. Deng L. Cai G. Liu X. Liu B. (2010). Characterization of a pathogenesis-related thaumatin-like protein gene TaPR5 from wheat induced by stripe rust fungus. *Plant Physiol.* 139 27–38. 10.1111/j.1399-3054.2009.01338.x 20059734

[B115] Wang Y. Zhou Z. Gao J. Wu Y. Xia Z. Zhang H. (2016). The mechanisms of maize resistance to *Fusarium verticillioides* by comprehensive analysis of RNA-seq data. *Front. Plant Sci.* 7:1654. 10.3389/fpls.2016.01654 27867390 PMC5096342

[B116] Warburton M. L. Williams W. P. (2014). Aflatoxin resistance in maize: what have we learned lately? *Adv. Bot.* 2014:352831 10.1155/2014/352831

[B117] Widstrom N. W. Wilson D. M. McMillian W. W. (1981). Aflatoxin contamination of preharvest corn as influenced by timing and method of inoculation. *Appl. Environ. Microbiol.* 42 249–251. 6792985 10.1128/aem.42.2.249-251.1981 PMC243998

[B118] Wild C. P. Miller J. D. Groopman J. D. (eds). (2015). *Mycotoxin Control in Low- and Middle Income Countries.* IARC Working Group Report No. 9 Lyon: International Agency for Research on Cancer. 27030861

[B119] Wilson R. A. Gardner H. W. Keller N. P. (2001). Cultivar-dependent expression of a maize lipoxygenase responsive to seed infesting fungi. *Mol. Plant Microbe Interact.* 14 980–987. 10.1094/MPMI.2001.14.8.980 11497470

[B120] Wisser R. J. Kolkman J. M. Patzoldt M. E. Holland J. B. Yu J. Krakowsky M. (2011). Multivariate analysis of maize disease resistances suggests a pleiotropic genetic basis and implicates a GST gene. *Proc. Natl. Acad. Sci. U.S.A.* 108 7339–7344. 10.1073/pnas.1011739108 21490302 PMC3088610

[B121] Wu S. Kriz A. L. Widholm J. M. (1994a). Molecular analysis of two cDNA clones encoding acidic class I chitinase in maize. *Plant Physiol.* 105 1097–1105. 7972490 10.1104/pp.105.4.1097 PMC159437

[B122] Wu S. Kriz A. L. Widholm J. M. (1994b). Nucleotide sequence of a maize cDNA for a class II, acidic beta-13-glucanase. *Plant Physiol.* 106 1709–1710. 10.1104/pp.106.4.1709 7846180 PMC159727

[B123] Wuren T. Toyotome T. Yamaguchi M. Takahashi-Nakaguchi A. Muraosa Y. Yahiro M. (2014). Effect of serum components on biofilm formation by *Aspergillus fumigatus* and other *Aspergillus* species. *Jpn. J. Infect. Dis.* 67 172–179. 10.7883/yoken.67.172 24858605

[B124] Xie Y. R. Chen Z. Y. Brown R. L. Bhatnagar D. (2010). Expression and functional characterization of two pathogenesis-related protein 10 genes from *Zea mays*. *J. Plant Physiol.* 167 121–130. 10.1016/j.jplph.2009.07.004 19682768

[B125] Yang D. L. Yang Y. He Z. (2013). Roles of plant hormones and their interplay in rice immunity. *Mol. Plant* 6 675–685. 10.1093/mp/sst056 23589608

[B126] Yilmaz A. Nishiyama M. Y. Jr. Fuentes B. G. Souza G. M. Janies D. Gray J. (2009). GRASSIUS: a platform for comparative regulatory genomics across the grasses. *Plant Physiol.* 149 171–180. 10.1104/pp.108.128579 18987217 PMC2613736

[B127] Zhang A. Zhang J. Ye N. Cao J. Tan M. Zhang J. (2010). ZmMPK5 is required for the NADPH oxidase-mediated self-propagation of apoplastic H_2_O_2_ in brassinosteroid-induced antioxidant defence in leaves of maize. *J. Exp. Bot.* 61 4399–4411. 10.1093/jxb/erq243 20693409 PMC2955750

[B128] Zhang J. Simmons C. Yalpani N. Crane V. Wilkinson H. Kolomiets M. (2005). Genomic analysis of the 12-oxo-phytodienoic acid reductase gene family of *Zea mays*. *Plant Mol. Biol.* 59 323–343. 10.1007/s11103-005-8883-z 16247560

[B129] Zhang Y. Cui M. Zhang J. Zhang L. Li C. Kan X. (2016). Confirmation and fine mapping of a major QTL for aflatoxin resistance in maize using a combination of linkage and association mapping. *Toxins* 8:E258. 10.3390/toxins8090258 27598199 PMC5037484

[B130] Zhu Y. Liu W. Sheng Y. Zhang J. Chiu T. Yan J. (2015). ABA affects brassinosteroid-induced antioxidant defense via ZmMAP65-1a in maize plants. *Plant Cell Physiol.* 56 1442–1455. 10.1093/pcp/pcv061 25941233

